# Impact of a new European regulations on functional food market – an overview

**DOI:** 10.1186/2045-7022-1-S1-S55

**Published:** 2011-08-12

**Authors:** Martinus Lovik

**Affiliations:** 1 Norwegian Institute of Public Health and Norwegian University of Technology and Science, Department of Environmental Immunology and Institute for Cancer Research and Molecular Medicine, Oslo and Trondheim, Norway

## 

Functional foods are defined as foods that provide health benefits in addition to their basic nutritional value. These additional health benefits form the basis for producers’ health claims on foods. Legislation demands that health claims should be based on and substantiated by generally accepted scientific evidence. The main topic of this presentation will be how the European Food Safety Authority (EFSA) approaches the issue of scientific substantiation of health claims on foods. Health claims can be categorized in different ways, but basically they will be of two types, namely those relating to maintaining or improving a biological function (‘function claims’), and those relating to reduction of a disease risk factor (‘risk reduction claims’). Disease itself can not be used as the formal end point, and claims on treatment or cure of disease can not be made. The target population of the health claim must be specified, and essential criteria are that the claim must be sufficiently specific to allow evaluation, the claim must represent a beneficial physiological effect, the food/food component for which the claim is made must be sufficiently well characterised, and last but not least there must be sufficient scientific evidence to substantiate the claim. All the criteria must be met. Scientific evidence is evaluated according to standard criteria for good science. The ongoing work in EFSA, with evaluation of some 4000 health claims, is an important step to secure that health claims on products in the marketplace are based on by sound, convincing scientific evidence, and that the claims will not soon have to be re-evaluated in light of new scientific data. Securing that claims are truthful, accurate and not misleading is expected over time to increase consumers’ trust in health claims on foods, and thereby supposedly also the use of products bearing health claims.

